# Allelic imbalance of multiple sclerosis susceptibility genes *IKZF3* and *IQGAP1* in human peripheral blood

**DOI:** 10.1186/s12863-016-0367-4

**Published:** 2016-04-14

**Authors:** Pankaj K. Keshari, Hanne F. Harbo, Kjell-Morten Myhr, Jan H. Aarseth, Steffan D. Bos, Tone Berge

**Affiliations:** Department of Neurology, Oslo University Hospital, Oslo, Norway; Institute of Clinical Medicine, University of Oslo, Oslo, Norway; Norwegian Multiple Sclerosis Registry and Biobank, Department of Neurology, Haukeland University Hospital, Bergen, Norway; KG Jebsen Centre for MS-research, Department of Clinical Medicine, University of Bergen, Bergen, Norway

**Keywords:** Allele specific expression, eQTL, Multiple sclerosis, *CD69*, *IKZF3*, *IQGAP1*

## Abstract

**Background:**

Multiple sclerosis is a chronic inflammatory, demyelinating disease of the central nervous system. Recent genome-wide studies have revealed more than 110 single nucleotide polymorphisms as associated with susceptibility to multiple sclerosis, but their functional contribution to disease development is mostly unknown.

**Results:**

Consistent allelic imbalance was observed for rs907091 in *IKZF3* and rs11609 in *IQGAP1*, which are in strong linkage disequilibrium with the multiple sclerosis associated single nucleotide polymorphisms rs12946510 and rs8042861, respectively. Using multiple sclerosis patients and healthy controls heterozygous for rs907091 and rs11609, we showed that the multiple sclerosis risk alleles at *IKZF3* and *IQGAP1* are expressed at higher levels as compared to the protective allele. Furthermore, individuals homozygous for the multiple sclerosis risk allele at *IQGAP1* had a significantly higher total expression of *IQGAP1* compared to individuals homozygous for the protective allele.

**Conclusions:**

Our data indicate a possible regulatory role for the multiple sclerosis-associated *IKZF3* and *IQGAP1* variants. We suggest that such *cis*-acting mechanisms may contribute to the multiple sclerosis association of single nucleotide polymorphisms at *IKZF3* and *IQGAP1*.

**Electronic supplementary material:**

The online version of this article (doi:10.1186/s12863-016-0367-4) contains supplementary material, which is available to authorized users.

## Background

Multiple sclerosis (MS) is a chronic inflammatory disorder of the central nervous system (CNS). Hallmarks of the disease are inflammatory demyelination and axonal loss in the CNS, resulting in neurological dysfunction, typically starting in early adulthood [1]. The cause of the disease is largely unknown, but genetic and environmental factors, as well as interaction of these contribute to disease development [2]. The strongest genetic factor in MS, the Human Leukocyte Antigen (*HLA*) gene region, was identified already in 1972 [3], and HLA-DRB1*15:01 displays the strongest genetic signal [4]. Through recent large genome - wide association studies (GWASs) followed up by genome-wide analyses of immune-related loci, more than 110 non-HLA genetic variants were shown to be associated with MS susceptibility [4, 5]. The majority of the MS-associated single nucleotide polymorphisms (SNPs) identified through GWAS are located in or near genes having a substantial role in the immune system [4, 6].

An important aim of human genetic research is identification of heritable variation in *cis*-regulatory elements for gene expression that might influence disease risk [7]. The majority (93 %) of the disease susceptibility variants identified through large-scale genetic screens such as GWAS and the immunochip project is located within non-coding regions of the genome [8]. For MS, an enrichment of disease-associated variants is observed in DNase hypersensitive sites in immune cells, especially for T and B cells [8, 9]. Genetic variants may therefore have an influence on the expression of target genes, and the MS-association of the polymorphisms identified through the genetic screens is likely to be the result of such regulatory properties. However, the functional implications of the genetic variants associated with MS are largely unknown. To address this, follow-up studies of these MS associated loci are needed to gain insights in the underlying molecular mechanisms. Analysis to unravel whether MS-associated SNPs exert *cis*-acting regulation is now an important next step in MS research. Such insights will contribute to improved understanding of disease aetiology and may allow development of novel biomarkers and therapies for MS.

The Genotype-Tissue Expression database (GTEx) is providing a valuable tool to investigate genotype-dependent gene regulation. The use of a great variety of tissues from human post-mortem donors allows comparing putative genotype-dependent gene regulation between at least 44 tissues in the publicly available pilot data [10]. Data from expression quantitative trait locus (eQTL) databases such as the GTEx is generally based on analyses in heterogeneous groups of individuals, however in specific diseases, gene regulation may be altered due to a disturbed homeostasis. For MS, it is proposed that whole blood composition and immune cell activity is different from healthy controls [11, 12]. In the current study, we have investigated the allele - specific expression (ASE) of MS-associated SNPs or proxies thereof in whole blood from a cohort of Norwegian MS patients. The unequal output of gene transcript, allelic imbalance (AI), has been established as a ubiquitous phenomenon that may underlie disease risk exerted by disease-associated SNPs [13]. AI may occur when the SNP-containing sequence is within a *cis*-regulatory region of a gene. A prerequisite to study putative effects on allelic output for disease associated SNPs is that the SNP itself, or a proxy SNP on the same haplotype as the susceptibility SNP, is transcribed. The per-allele expression levels for a transcribed SNP can then be determined in individuals heterozygous for the studied SNP.

Here, we investigate three SNPs in MS associated regions for the presence of AI; (i) the MS-associated SNP rs11052877, which lies in the coding region of *CD69*, (ii) the rs907091 SNP in the coding region of *IKZF3* and in strong linkage disequilibrium (LD) with the MS-associated rs12946510 and (iii) the rs11609 SNP located in the coding region of *IQGAP1*and in strong LD with the MS-associated rs8042861. Although the data from the GTEx database is extensive, there are examples of genotype-expression correlations not detected. Interestingly, GTEx reports no effect of rs907091 genotype on *IKZF3* expression, whereas Fahr and colleagues [14] refer to rs907091 as an eQTL for *IKZF3* in lymphoblastoid cell lines [15]. Furthermore, rs11609 in *IQGAP1* is reported as an eQTL for *IQGAP1* in a wide variety of tissues, but not in whole blood, whereas no SNPs are reported as eQTLs for *CD69* in any of the tissues (GTEx, accessed March 2016). In the current analyses, ASE measurements for three SNPs in these three genes (*CD69*, *IKZF3* and *IQGAP1*) were performed in whole blood samples from Norwegian MS patients and healthy controls.

## Methods

### Subjects, sample collection and reverse transcription of RNA

Blood samples were collected for DNA (EDTA tubes, Greiner Bio-One, Frickenhausen, Germany) and RNA isolation (Tempus tubes, Applied Biosystems, Foster City, CA, USA) from 140 MS patients collected in the Norwegian multiple sclerosis registry and biobank [16] and 46 healthy controls (14 for RNA extraction) recruited among hospital employees. The MS patients are representative of the Norwegian MS patient population [17]. DNA isolations were performed using DNA isolation columns, and RNA was extracted from Tempus tubes with the Tempus Spin RNA Isolation Kit protocol (Applied Biosystems), including DNase treatment during extraction. The concentration of all DNA and RNA samples was assessed by a nanodrop 2000c spectrophotometer (Thermo Fisher Scientific Inc., Madison, WI, USA). An RNA integrity number above 7.0 of a random set of these RNA samples was verified using the Agilent 2100 Bioanalyzer (Agilent Technologies, Santa Clara, CA, USA). For DNA samples that were used for sensitivity analyses of assays for ASE analyses (see below), the concentration was measured by Qubit Broad Range DNA spectroscopy (Qubit, Life Technologies, Carlsbad, CA, USA). According to manufacturers’ protocol, 200 ng RNA was reverse transcribed (RT) with Maxima First Strand complementary DNA (cDNA) synthesis Kit for quantitative polymerase chain reaction (qPCR) (Thermo Scientific, Pittsburgh PA, USA) in a 20 μl reaction.

### Single nucleotide polymorphisms included for allele-specific expression analysis

We used the online tools SNAP for identifying proxy SNPs for the MS associated SNPs [18] and SNPper [19] for determining which of these proxies were transcribed for all 110 non-HLA MS-associated SNPs recently reported [4, 5]. Three genes, which had a transcribed MS-associated SNP or transcribed proxy for an MS-associated SNP were selected for ASE analyses; *CD69*, with the MS-associated SNP rs11052877 in its coding region, and *IKZF3* and *IQGAP1*, where rs907091 and rs11609, respectively, are in strong LD (r^2^ ≥ 0.8) with MS-associated SNPs. To verify that the SNP-containing regions were expressed in whole blood, PCRs followed by agarose gel electrophoresis were done on cDNA generated from whole blood. Primer sequences are provided in Additional file 1: Table S1.

### Genotyping

Genotyping of rs11052877, rs907091, rs11609, rs12946510 and rs8042861 in DNA from the MS patients and healthy controls included those for the Western blot analyses (rs11609 only) was done with TaqMan genotyping assays (C_32169538_10, C_7452266_20, C_26774507_10, C_31651862_10 and C_12091833_10 respectively (Applied Biosystems)). The genotyping reactions were carried out in 5 μl using 12 ng of genomic DNA (gDNA), 0.125 μl 40x TaqMan assay and 2.5 μl TaqMan genotyping master mix in a MicroAmp Optical 384 well reaction plate (Applied Biosystems) on a ViiA7 Real-Time PCR system (Applied Biosystems). Data were analysed by the sequence detection system (SDS) v. 2.3 (Applied Biosystems). Healthy control genotypes for the ASE analyses of ten of the samples were assessed previously [20], whereas the remaining four samples were genotyped as described above.

### Sensitivity assessment of allele-specific expression

To assess the sensitivity of the allele-specific qPCR, gDNA homozygous for either allele of the corresponding SNPs were mixed in ratios of 4:1, 2:1, 1.5:1, 1.25:1, 1:1, 1:1.25, 1:1.5, 1:2 and 1:4. The ratios of allele-specific signals of these mixtures were quantified by real-time qPCR using TaqMan genotyping assays in TaqMan Universal Master Mix II, without UNG (Applied Biosystems), while including a heterozygous gDNA sample as a 1:1 ratio control of the alleles (Additional file 2: Figure S1). The samples were run in triplicate in a MicroAmp Optical 384 well reaction plate (Applied Biosystems) on ViiA7 Real-Time PCR system (Applied Biosystems), and data were analysed by SDS v. 2.3 (Applied Biosystems).

### Allele-specific expression

For each cDNA sample from heterozygous donors, five replicate real-time qPCR measurements were performed using 5.66 ng of cDNA in a 10 μl reaction additionally containing 0.25 μl 40x Primer Probe mix (Applied Biosystems), 5 μl TaqMan Universal master mix II, without UNG (Applied Biosystems) and 4.15 μl RNase-free water (Qiagen, Hilden, Germany). Genomic DNA (50 ng) from all heterozygous individuals was used to determine the technical difference in signals between alleles at an equal allele presence. The gDNA and cDNA samples were assayed using the same genotyping TaqMan primers and probes for the respective assays. All cDNA measurements were normalized against this technical difference (see data analyses and statistics below). Each plate included a negative control without cDNA and a no–RT control. The PCR reactions were performed in a MicroAmp optical 384 well reaction plate (Applied Biosystems) on the ViiA7 Real-Time PCR system (Applied Biosystems), and data were analysed by SDS v. 2.3 software (Applied Biosystems).

### Quantitative real-time polymerase chain reaction of homozygous samples

For each homozygous sample, cDNA generated from 6.52 ng RNA was assayed in 10 μl final volume containing 0.5 μl of 20x Primer Probes (*IKZF3*: Hs00232635_m1 or *IQGAP1*: Hs00896595_m1, Applied Biosystems), 5 μl TaqMan Gene expression Master mix (Applied Biosystems) and 3.5 μl RNase-free water (Qiagen). A negative control without template and no–RT control were included in each plate. The reactions were performed in duplicates in a MicroAmp optical 384 well reaction plate (Applied Biosystems) on the ViiA7 Real-Time PCR system (Applied Biosystems), and data were analysed by SDS v. 2.3 software (Applied Biosystems). A standard curve was prepared from whole blood RNA from MS cases with a 1:2 fold dilution series (33.33-0.26 ng/μl). Gene expression was normalized relative to the housekeeping genes *18S rRNA* (4319413E), *TBP* (4326322E), and *GAPDH* (Hs03929097_g1). *18S rRNA* was selected as the preferential reference gene given its low variance in Ct between the different samples (data not shown).

### Peripheral blood mononuclear cells collection, cell lysis and Western blot analyses

Whole blood samples from 32 healthy donors were collected among hospital employees and peripheral blood mononuclear cells were isolated by Lymphoprep (Axis Shield, Dundee, Scotland). Cells were resuspended in reducing SDS-loading buffer, sonicated and heated at 95 °C for 5 min. Proteins from 250,000 cells were separated by SDS-polyacrylamide gel electrophoresis using pre-made Criterion gels (BioRad, Hercules, CA, USA) and transferred to polyvinylidene fluoride membrane (BioRad) using a Hoefer Semi-Phor Semi-Dry transfer unit (Amersham Biosciences, Buckinghamshire, UK). The membrane was blocked in 3 % skimmed milk in Tris-buffered saline (TBS, pH 7.4) containing 0.1 % Tween-20 (Sigma Aldrich Corp., St Louis, MO, USA) (TBS-T) before incubation with antibodies, rabbit anti-IQGAP1 (ab133490, Abcam, Cambridge, UK) or mouse anti-GAPDH (6C5, sc-32233, Santa Cruz Biotechnology, Dallas, TX, USA). Bound antibodies were visualized by incubation with secondary horseradish peroxidase-conjugated goat anti-rabbit IgG or goat anti-mouse IgG (Jackson ImmunoResearch Laboratories Europe Ltd., Suffolk, UK) and ECL prime Western blotting detection reagent (GE Healthcare, Oslo, Norway ) and the ChemiDoc Touch Imaging System (BioRad). Densiometry of the Western blots was analysed by the ImageJ software [21].

### Data analyses and statistics

Per donor in the ASE measurements, outlier values were excluded after inspection of box plots using SPSS (IBM SPSS Statistics v21.0, Chicago, IL, USA). Initially, measurements with extreme values were excluded, followed by generation of new box plots for the remaining data. Values marked as outlier values in the newly generated box plots were also excluded before analyses (for details on excluded measurements, see Additional file 3: Table S2). For each heterozygous donor, the relative allelic expression of the two alleles was expressed as delta cycle threshold (ΔCt) = Ct (Allele2, FAM)—Ct (Allele1, VIC). To account for technical differences between the used fluorophores, the ΔCt was normalized to the mean ΔCt of all gDNA (normalized ΔCt (nΔCt) = ΔCt_cDNA (per sample)_ -ΔCt_gDNA (mean all samples)_). For each assessed SNP, a two-tailed, unpaired Student’s *t*-test was used to identify significant differences of the nΔCt per sample using the gDNA measurements grouped as the reference group. To test for differences on a global level, a two-tailed, unpaired Student’s *t*-test was performed on all cDNA pooled against all gDNA pooled. Association between gene or protein expression and genotype was assessed by two-sided Mann–Whitney *U*-test using GraphPad Prism 6 (GraphPad Software, Inc., San Diego, CA, USA). *P*-values ˂0.05 were considered significant.

## Results

### Patients and controls

A total of 140 MS patients were available for the study, of whom 92 were heterozygous for at least one of the three SNPs studied; i.e. rs11052877 (*CD69*), rs907091 (*IKZF3*) and rs11609 (*IQGAP1*), and therefore included in the analyses. In Table 1 we summarize the characteristics of the patients and the healthy controls used in the AI measurements such as the male to female ratio, age at onset and fraction of oligoclonal band positive patients. The included patient group was representative of the Norwegian MS patient population [17]. Treatment data for the patients at the time of blood drawing was not available.

**Table 1:** Clinical and demographic information about the included MS patients and healthy controls

			Healthy controls			
	MS patients		ASE analyses		Western blot analyses	
Variable	*n*=		*n*=		*n*=	
Male : Female ratio	92	1 : 3.4	14	1 : 4.7	32	1 : 1.5
Age in years, mean (range) at sampling	92	50 (19–80)	14	39 (22–57)	32	37 (21–68)
Age in years, mean (range) at disease onset	89	35 (14–60)				
Disease course at onset, fraction of relapsing-remitting MS	86	0.87				
Oligoclonal bands, positive fraction	68	0.91				

### Selection of single nucleotide polymorphisms for allele-specific expression of multiple sclerosis candidate genes

From the list of 110 non-HLA MS-associated SNPs [4, 5], we selected three SNPs for ASE analyses, based on the gene containing a transcribed SNP or suitable proxy SNP and the possible biological role of the gene in the immune system. The MS-associated SNP rs11052877 is located in a transcribed region of *CD69*, whereas the remaining transcribed SNPs analysed; rs907091 in *IKZF3* and rs11609 in *IQGAP1*, are in high LD with the MS-associated SNPs. The r^2^ (D’) of rs907091 to rs12946510 is 0.80 (0.90), whereas the r^2^ (D’) of rs11609 to rs8042861 is 0.86 (0.93).

### Consistent allelic imbalance is observed for rs907091 in *IKZF3* and rs11609 in *IQGAP1*

Among the 92 MS patients that were heterozygous for at least one of the three SNPs studied, 58 patients were heterozygous for rs11052877 (*CD69*), 30 patients were heterozygous for rs907091 (*IKZF3*) and 61 were heterozygous for rs11609 (*IQGAP1*). For each SNP, these samples were analysed for ASE by quantification of the alleles in cDNA generated from whole blood.

**Fig. 1:**
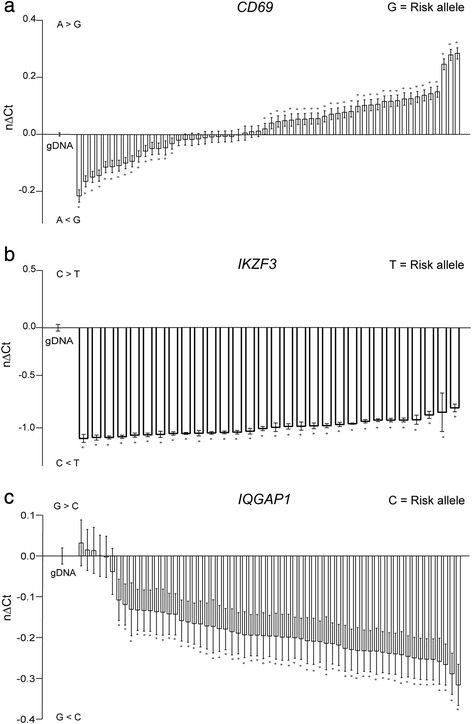
Consistent allelic imbalance is observed for rs907091 in *IKZF3* and rs11609 in *IQGAP1*. ASE analyses for (**a**) rs11052877 in *CD69*, (**b**) rs907091 in *IKZF3*, and (**c**) rs11609 in *IQGAP1* were performed in samples from whole blood from MS patients heterozygous for the indicated SNPs. Each bar represents five replicate measurements. Data are presented as the normalized change in Ct between the two alleles (nΔCt). nΔCt values above zero represents lower expression of the MS risk allele, whereas nΔCt values below zero represent higher expression of the MS risk allele. Error bars represent the standard error of the mean. Unpaired Student’s t-tests were used to compare each column with the gDNA measurement, *P*-values <0.05 were considered significant and are indicated with an asterisk. A > B = allele A expressed higher than B, A < B = allele A expressed lower than B

For the rs11052877 SNP in *CD69*, 52 % of the samples carrying the MS risk allele displayed lower expression of the MS risk allele, whereas 26 % of the samples displayed higher expression and the remaining 22 % of the samples displayed no significant AI (Fig. 1a). This shows that there is no consistent *cis*-regulatory mechanism for *CD69* associated with this SNP. Contrary to that, the transcript of the haplotype containing the MS risk allele at rs907091 in *IKZF3*, showed consistently higher expression level in all MS samples (Fig. 1b), whereas the transcript of the haplotype containing the MS risk allele at rs11609 in *IQGAP1* was higher in the majority (90 %) of MS samples (Fig. 1c). For rs907091 in *IKZF3*, this difference corresponded to 1.17 in nΔCt value and to an expression ratio of the MS risk allele of 2.25:1. For rs11609 in *IQGAP1*, the nΔCt in the significant samples was 0.202, which corresponds to an expression ratio of the risk allele versus non-risk allele of 1.15:1. When we compared all cDNA as a group against all gDNA as the reference group, the significant differences in AI between these two groups persisted for *IKZF3* as well as *IQGAP1*.

Since an incomplete LD (r^2^ = 0.86, D’ = 0.93) exists between the studied SNP rs11609 and the reported MS associated SNP rs8042861 [5] in *IQGAP1*, we analysed whether the lack of AI for rs11609 in a small proportion of the samples could be due to absence of the MS-risk allele for these samples. However, all samples except one were also heterozygous for rs8042861 (data not shown), eliminating the possibility that absence of AI in the minority of the samples could be ascribed to a different genotype at the MS-risk SNP.

To unravel whether the consistent AI observed for *IKZF3* could be attributed to the SNP most strongly associated to MS in this gene (rs12946510, which is in high, but incomplete LD with rs907091 (r^*2*^ = 0.80, D’ = 0.90)), the samples were also analysed for double heterozygosity. We found that five of the samples were homozygous for the MS top hit rs12946510. This indicates that the observed AI is not driven by the genotype of the MS top hit rs12946510 in whole blood.

To exclude that the observed AI for rs907091 and rs11609 was limited to MS patients, we investigated ASE also in a smaller set of samples from healthy controls. We observed AI similar to the MS patients’ samples indicating that the observed AI is independent of disease status (Additional file 4: Figure S2).

Altogether, these data indicate that the studied SNPs in *IKZF3* and *IQGAP1* are located in a *cis*-regulatory element, or that these SNPs mark a functional *cis*-regulatory element that impacts the per-allele transcript abundance in whole blood, independent of disease status.

### Samples from homozygous carriers of the risk allele at rs11609 display higher *IQGAP1* expression

**Fig. 2:**
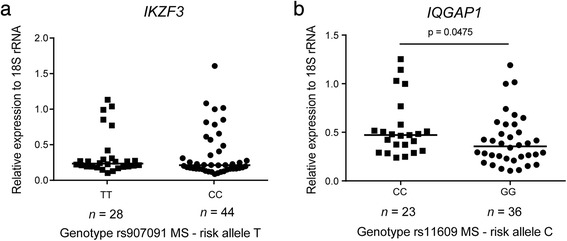
Samples from homozygous carriers of the risk allele at rs11609 display higher *IQGAP1* expression. Expression of *IKZF3* and *IQGAP1* relative to *18S rRNA* in samples from MS patients genotyped for (**a**) rs907091 in *IKZF3* (TT (risk): *n* = 28; CC (protective): *n* = 44) and (**b**) rs11609 *IQGAP1* (CC (risk): *n* = 23; GG (protective): *n* = 36). Samples from individuals homozygous for the risk allele were compared with samples from individuals homozygous for the protective allele with two-tailed Mann-Whitney *U*-test. *P*-values <0.05 were considered significant, only the significant *p*-value is provided in the graph

To follow up the consistent AI of *IKZF3* and *IQGAP1* observed in heterozygous individuals, we selected the samples that were homozygous for either allele of rs907091 (*IKZF3*) and rs11609 (*IQGAP1*) to determine whether the difference in allelic expression results in a difference in overall gene expression. We did not observe significant differences in *IKZF3* gene expression between these two groups of homozygous for either the risk allele or protective allele (Fig. 2a). However, for *IQGAP1*, a significant genotype dependent expression was identified with higher expression in samples homozygous for the risk allele (Fig. 2b), in line with our ASE analyses described above. To further investigate whether higher *IQGAP1* for the MS risk allele results in a higher IQGAP1 protein level, we measured this expression by Western blotting in lysates from peripheral blood mononuclear cells from healthy individuals homozygous for either the risk or protective rs11609 allele at *IQGAP1*. Although we observe a slightly higher IQGAP1 expression in samples homozygous for the risk allele, this did not reach significance (Additional file 5: Figure S3).

## Discussion

Recent large-scale genotyping studies in MS have revealed more than 110 genetic variants associated with MS [4, 5], however, the functional impact of the majority of these SNPs remains unknown and needs to be determined experimentally. To this end, we investigated the ASE of three transcribed SNPs on MS susceptibility haplotypes in whole blood of MS patients and show AI for the SNPs rs907091 and rs11609, which are transcribed in *IKZF3* and *IQGAP1*, respectively. Furthermore, we observed a significantly higher level of *IQGAP1* gene expression for samples homozygous for the allele with a relatively higher expression in the ASE measurements, possibly indicating that the allelic imbalance persists beyond the relative expression of the alleles. Given this observation, a relative higher expression of *IQGAP1* for carriers of the minor allele might contribute to an increase in MS susceptibility.

Allele-specific qPCR is a sensitive tool for evaluation of *cis*-acting regulatory polymorphisms and can directly detect differences in allelic output when assessed in heterozygous carriers. By examining the relative expression levels of two alleles within the same biological sample, variations introduced by the environment or differences in physiological background of the individuals enrolled in the study were minimized. AI measurements are relatively robust for detecting and quantifying variations in allelic expression. However, the AI observed in the MS cases for *IKZF3* and *IQGAP1* was also observed in healthy controls, indicating that MS itself does not impact the AI of these genes.

It is important to note that the overall expression of a gene can be influenced by multiple regulatory mechanisms. Here we have a specific focus on those genetic loci that were shown to have an association to MS. The rs11609 SNP in *IQGAP1* has been identified as an eQTL for *IQGAP1* in many tissues (for instance mammary, adipose, thyroid, stomach and skin tissue), however not in whole blood. In our study, we observe relatively higher allelic expression of the MS-risk allele at rs11609, which is consistent with higher overall gene expression of *IQGAP1* in samples homozygous for the risk allele. This is similar to data from other tissues in the GTEx data where rs11609 is reported as an eQTL with higher expression for carriers of the minor allele. Our data indicate that also in whole blood, the haplotype on which rs11609 resides has *cis*-regulatory properties affected by genetic variation. We found double heterozygosity for all samples except one for rs8042861 and rs11609 in *IQGAP1*. The observed AI for rs11609 may originate from different mechanisms of *IQGAP1* gene regulation. Interestingly, rs11609 is situated in an open chromatin region (genome.ucsc.edu), making it a plausible candidate for influencing gene expression. However, there are several SNPs in high LD with the MS associated rs8042861 SNP that are within transcription factor binding sites (genome.ucsc.edu) and thereby might be the causal SNP mediating the observed effect. Indeed, it has been suggested that for most of the complex disease-associated SNPs, the underlying SNP is predicted to be located within the LD block of the associated SNP [14]. Further studies of genetic variation present in this region of interest for functional properties are necessary to better discern among these possibilities. The exact molecular mechanism of how higher *IQGAP1* expression may contribute to increased MS risk is unclear and warrants more investigation. IQ Motif Containing GTPase Activating Protein 1 (IQGAP1) is a ubiquitously expressed protein, belonging to the scaffolin-family, and has emerged as a critical regulator of several signalling pathways in a variety of cell types, including immune cells, where it plays important roles for cytoskeleton-mediated processes [22]. IQGAP1 has shown to be instrumental for leukocyte chemotaxis and natural killer (NK)-cell cytotoxicity [23, 24]. Higher IQGAP1 expression, as observed from MS risk loci, would lead to aberrant leukocyte cell migration and NK cell activity, and could thereby contribute to MS disease. Furthermore, defects of the IQGAP1-interacting protein, Rap1b, result in multiple NK and B-cell dysfunctions [25, 26], indicating that a change in IQGAP1 expression in those immune cells could also have an impact for MS development. Although we observed higher IQGAP1 protein expression in samples homozygous for the risk allele, this did not reach statistical significance. To reach 80 % power to detect a difference of this magnitude with this standard deviation, a sample size of at least 43 in each group would be required (Additional file 6).

In agreement with the GTEx database (accessed March 2016), we did not observe consistent allelic imbalance for the *CD69* gene, indicating that the SNP association to MS may not be attributable to *cis*-regulatory properties in whole blood. Whether there are such *cis*-regulatory properties for this SNP in more specialized cell sub-types within the whole blood or other cells relevant to MS remains to be investigated. However, in line with our results, recent bioinformatic analyses indicated that the genotype of the MS associated rs11052877 SNP does not affect any transcription factor DNA binding motifs [27].

The rs907091 SNP in *IKZF3* has been shown to be an eQTL influencing the expression or stability of the gene transcript in lymphoblastoid cell lines [14, 15], but not in whole blood (GTEx, accessed March 2016). The consistent AI observed for rs907091 indicates that this SNP potentially has eQTL properties in whole blood. We did not observe differences of overall *IKZF3* gene expression between the homozygous carriers of the minor and major allele, indicating that the eQTL properties may be most pronounced in homogeneous samples. Alternatively, our study may have a limited sample size and therefore lack the power to detect such differences in whole blood. Furthermore, the blood samples used in this study are from an MS patient population at different disease stages and using different treatments. This heterogeneity within the sampled patients could contribute to the lack of differential gene expression in samples sorted on different genotypes.

It has previously been suggested that most disease variants exert subtle and highly context-dependent effects on gene regulation [14], therefore we cannot exclude that using a comparable number of samples from specific immune cell subsets could detect *IKZF3* expression differences depending on the SNP genotype. The SNP rs907091 is located in the 3’ untranslated region of the *IKZF3* gene encoding AIOLOS, an Ikaros transcription factor involved in regulation of lymphocyte development [28]. *IKZF3* deficiency in mice mainly affects B cell function [29], giving rise to hyperactive immature B cell precursors [30, 31], and decreased peritoneal, marginal and recirculating B cells [30, 32, 33]. An increase in *IKZF3* expression in B cells might lead to aberrant B-cell responses that could play a role for MS development. Since there is a lack of complete double heterozygosity between the AI SNP and the MS top-hit SNP rs12946510 in *IKZF3*, the consistent AI observed for rs907091 cannot be solely attributed to the genotype of the MS-associated SNP. The mechanisms through which the rs12946510 might have functional consequences for MS development need further investigations. Moreover, the probabilistic identification of causal SNPs fine mapping algorithm from the Broad Institute [14] indicates that a large array of SNPs may be underlying the identified MS association signal, including rs907091, without a clear indication for the actual functional SNP at this locus. Therefore, the *IKZF3* AI can be driven by multiple SNPs without any of these having a probability above 20 % of being the true functional SNP. Previous experimental studies have indicated that polymorphisms in *IKZF3* influence susceptibility for development of several autoimmune disorders in addition to MS [14, 34–38]. Further investigations into how perturbation of the investigated genes in whole blood or in more specific cell subtypes are needed to further elucidate how the SNPs *cis*-regulation may contribute to MS risk.

## Conclusions

This study showed a molecular mechanism, which might contribute to the MS-association of two SNPs. We identified differences in allelic output and/or overall gene expression for rs907091 in *IKZF3* and rs11609 in *IQGAP1*, which are both in high LD with MS-associated SNPs. Additional studies into the functional properties of these SNPs and the impact on the protein expression in specific cell types that are present in whole blood may identify which cells drive the association of these SNPs to MS development. Furthermore, since these SNPs reside in large LD blocks that could harbour one or more true functional variants, additional investigations to identify the true functional variations are warranted.

### Ethics (and consent to participate)

The Regional Committee for Medical and Health Research Ethics South East, Norway, approved this study. Signed informed consent was obtained from all study participants.

### Consent to publish

Not applicable.

### Availability of data and materials

The datasets supporting the conclusions of this article are included in Additional file 7.

## Additional files

Additional file 1: Table S1. PCR primers used to verify gene expression of SNP-containing regions of the indicated genes in whole blood. (PDF 133 kb) Additional file 2: Figure S1. Assay sensitivity testing for allele-specific expression with known ratios of alleles. Allele-specific qPCR was performed on mixtures of known ratios of gDNA obtained from samples homozygous for either allele of rs11052877, rs907091 or rs11609, respectively. Mixtures were prepared with allele ratios of 4:1, 2:1, 1.5:1, 1.25:1, 1:1, 1:1.25, 1:1.5, 1:2 and 1:4. As control for a 1:1 allele ratio, a heterozygous sample (H) was included for each assay. Data is expressed as the mean of three measurements and error bars represent the standard error of the mean. (TIF 1553 kb) Additional file 3: Table S2. Details of excluded measurements per investigated gene. (PDF 82 kb) Additional file 4: Figure S2. Allele-specific expression analyses of *IKZF3* and *IQGAP1* in samples from healthy controls. Allele-specific expression of the genes was normalised to the mean of all genomic DNA for (a) rs907091 in *IKZF3* and (b) rs11609 in *IQGAP1*. Each bar represents five replicate measurements. Data are presented as the normalized change in Ct between the two alleles (nΔCt). nΔCt values above zero represent lower expression of the MS risk allele, whereas nΔCt values below zero represents higher expression of the MS risk allele. Error bars represent the standard error of the mean. A two-tailed unpaired Student’s t-test was used to compare each column with the gDNA measurement, *P*-values <0.05 are indicated with *. A > B = allele A expressed higher than B, A < B = allele A expressed lower than B. (TIF 84 kb) Additional file 5: Figure S3. IQGAP1 protein expression in peripheral blood mononuclear cells from healthy controls. (a) Whole-cell lysates from peripheral blood mononuclear cells from healthy controls genotyped for rs11609 in *IQGAP1* (CC (risk): *n* = 6; GG (protective): *n* = 9) were immunoblotted with indicated antibodies. (b) Bands were quantified and normalized with GAPDH as described in Materials and Methods. The graph shows the mean with standard error of the mean. A two- sided Mann-Whitney *U*-test was performed to compare the groups. (TIF 1357 kb) Additional file 6: Power analyses of IQGAP1 protein expression. (PDF 141 kb) Additional file 7: Raw dataset supporting the conclusions of this article. (XLSX 31 kb)

## Abbreviations

ASE: allele-specific expression
*CD69*: cluster of differentiation 69
cDNA: complementary DNA
CNS: central nervous system
Ct: cycle threshold
eQTL: expression quantitative trait loci
gDNA: genomic DNA
GWAS: genome - wide association study
*HLA*: human leukocyte antigen
*IKZF3*: IKAROS family zinc finger 3
*IQGAP1*: IQ motif containing GTPase activating protein 1
LD: linkage disequilibrium
MAF: minor allele frequency
MS: multiple sclerosis
NK: natural killer
qPCR: quantitative polymerase chain reaction
RT: reverse transcription
SNPs: single nucleotides polymorphisms
