# Supplementary material for: Allelic imbalance of multiple sclerosis susceptibility genes IKZF3 and IQGAP1 in human peripheral blood
Source: BMC Genet. 2016 Apr 14;17:59. doi: 10.1186/s12863-016-0367-4 (PMC4832550; doi:10.1186/s12863-016-0367-4)
Supplement: Additional file 1: Table S1. — PCR primers used to verify gene expression of SNP-containing regions of the indicated genes in whole blood. (PDF 133 kb) [file 12863_2016_367_MOESM1_ESM.pdf]

Additional file 1: Table 1. PCR primers used to verify gene expression of SNP-containing regions of the indicated genes in whole blood.

Gene	Sense primers (5'-3')	Antisense primers (5'-3')
CD69	GCAACCTTTGGATGCACTTT	GCTCCCTGAGTTTAAGGGATT
IKZF3	TTTCCCATCAGGGACTTGAA	GTCAGCTGGTCAGCTCTTCC
IQGAP1	TTTGACCTTCTCCTCGACCA	GATACAAAGGCAACTATGTGCAG
