# Supplementary figures and images for: Allelic imbalance of multiple sclerosis susceptibility genes IKZF3 and IQGAP1 in human peripheral blood
Source: BMC Genet. 2016 Apr 14;17:59. doi: 10.1186/s12863-016-0367-4 (PMC4832550; doi:10.1186/s12863-016-0367-4)

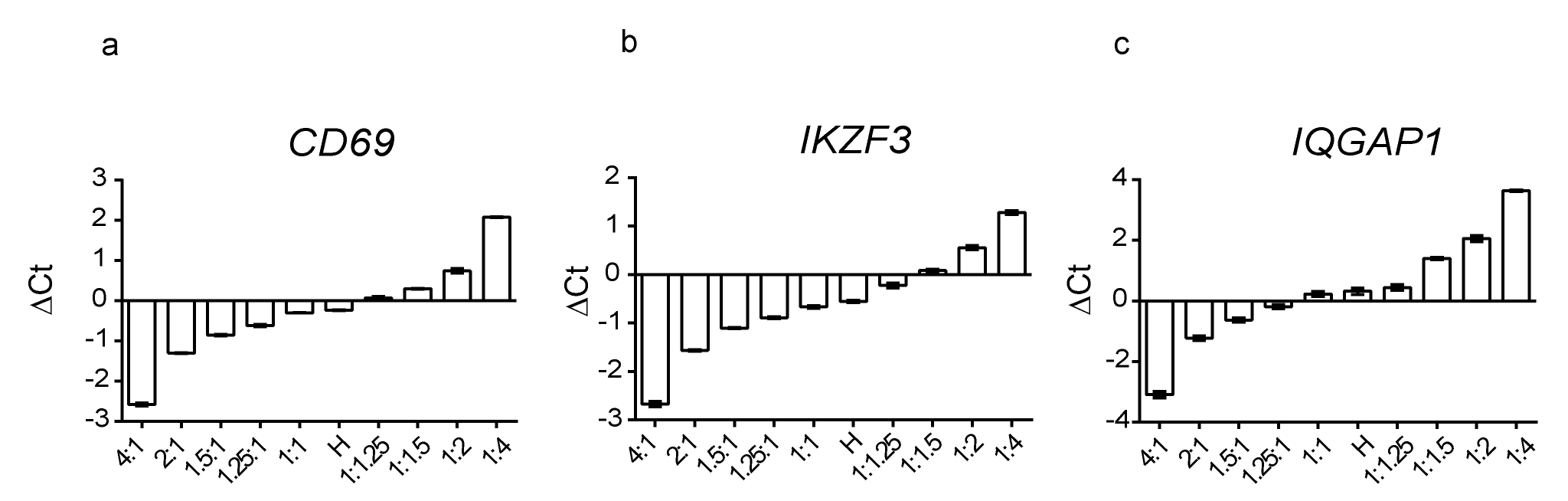

Supplement: Additional file 2: Figure S1. — Assay sensitivity testing for allele-specific expression with known ratios of alleles. Allele-specific qPCR was performed on mixtures of known ratios of gDNA obtained from samples homozygous for either allele of rs11052877, rs907091 or rs11609, respectively. Mixtures were prepared with allele ratios of 4:1, 2:1, 1.5:1, 1.25:1, 1:1, 1:1.25, 1:1.5, 1:2 and 1:4. As control for a 1:1 allele ratio, a heterozygous sample (H) was included for each assay. Data is expressed as the mean of three measurements and error bars represent the standard error of the mean. (TIF 1553 kb) [file 12863_2016_367_MOESM2_ESM.tif]

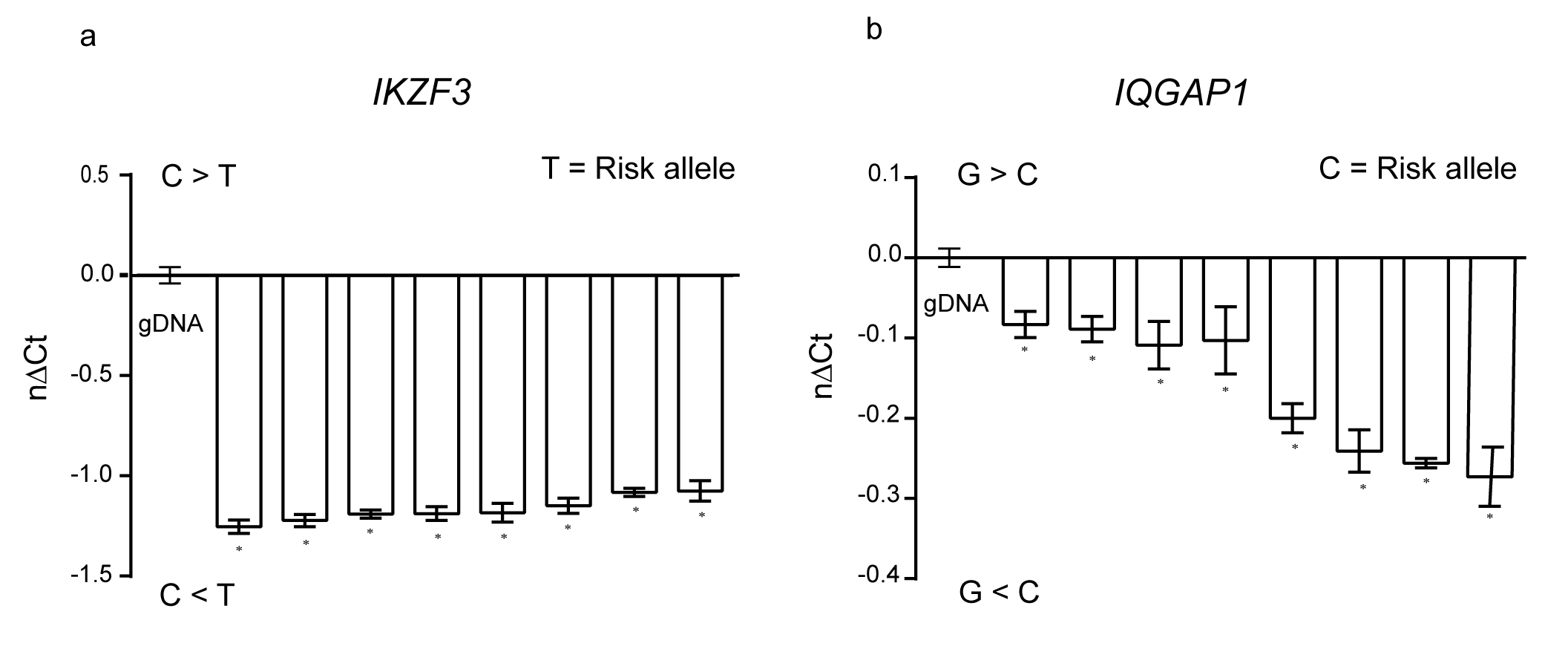

Supplement: Additional file 4: Figure S2. — Allele-specific expression analyses of IKZF3 and IQGAP1 in samples from healthy controls. Allele-specific expression of the genes was normalised to the mean of all genomic DNA for (a) rs907091 in IKZF3 and (b) rs11609 in IQGAP1. Each bar represents five replicate measurements. Data are presented as the normalized change in Ct between the two alleles (nΔCt). nΔCt values above zero represent lower expression of the MS risk allele, whereas nΔCt values below zero represents higher expression of the MS risk allele. Error bars represent the standard error of the mean. A two-tailed unpaired Student’s t-test was used to compare each column with the gDNA measurement, P-values <0.05 are indicated with *. A > B = allele A expressed higher than B, A < B = allele A expressed lower than B. (TIF 84 kb) [file 12863_2016_367_MOESM4_ESM.tif]

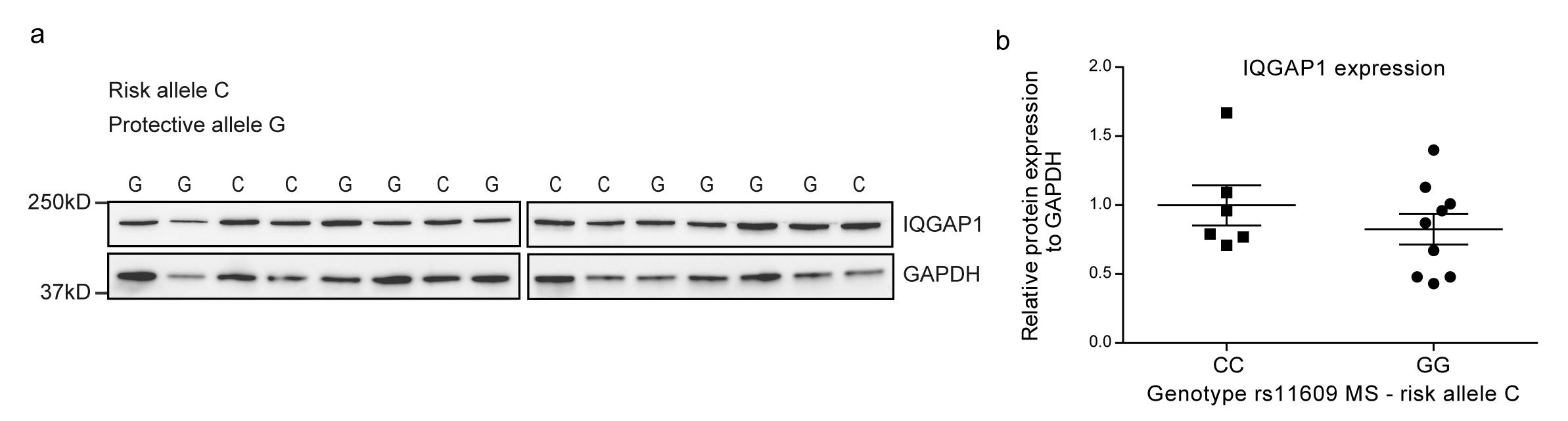

Supplement: Additional file 5: Figure S3. — IQGAP1 protein expression in peripheral blood mononuclear cells from healthy controls. (a) Whole-cell lysates from peripheral blood mononuclear cells from healthy controls genotyped for rs11609 in IQGAP1 (CC (risk): n = 6; GG (protective): n = 9) were immunoblotted with indicated antibodies. (b) Bands were quantified and normalized with GAPDH as described in Materials and Methods. The graph shows the mean with standard error of the mean. A two- sided Mann-Whitney U-test was performed to compare the groups. (TIF 1357 kb) [file 12863_2016_367_MOESM5_ESM.tif]
