# Supplementary material for: Allelic imbalance of multiple sclerosis susceptibility genes IKZF3 and IQGAP1 in human peripheral blood
Source: BMC Genet. 2016 Apr 14;17:59. doi: 10.1186/s12863-016-0367-4 (PMC4832550; doi:10.1186/s12863-016-0367-4)
Supplement: Additional file 3: Table S2. — Details of excluded measurements per investigated gene. (PDF 82 kb) [file 12863_2016_367_MOESM3_ESM.pdf]

**Additional file 3: Table 2.** Details of excluded measurements per investigated gene.

| Gene | Samples available | gDNA excluded values (in samples) | cDNA excluded values (in samples) |
|---|---|---|---|
| *CD69* | 58 | 1 (1) | 16/290 (12) |
| *IKZF3* | 30 | 1 (1) | 6/150 (6) |
| *IQGAP1* | 61 | 6 (6) | 22/305 (22) |
