# Supplementary material for: Allelic imbalance of multiple sclerosis susceptibility genes IKZF3 and IQGAP1 in human peripheral blood
Source: BMC Genet. 2016 Apr 14;17:59. doi: 10.1186/s12863-016-0367-4 (PMC4832550; doi:10.1186/s12863-016-0367-4)
Supplement: Additional file 6: — Power analyses of IQGAP1 protein expression. (PDF 141 kb) [file 12863_2016_367_MOESM6_ESM.pdf]

Additional file 6: Power analyses of IQGAP1 protein expression.

Power analysis using the "stats" package in R (version 3.2.1) indicates that to have 80% power to detect a putative real significant difference of the magnitude 0.17 seen in this group (standard deviation 0.276), we would need at least 43 people of each genotype. The most likely scenario is however that there is no detectable difference in protein expression in peripheral blood mononuclear cells, even though the gene expression can be different.
